# Tuning atomic-scale mixing of nanoparticles produced by atmospheric-pressure spark ablation†

**DOI:** 10.1039/d3na00152k

**Published:** 2023-08-23

**Authors:** Klito C. Petallidou, Pau Ternero, Maria E. Messing, Andreas Schmidt-Ott, George Biskos

**Affiliations:** a Climate and Atmosphere Research Centre, The Cyprus Institute 2121 Nicosia Cyprus g.biskos@tudelft.nl g.biskos@cyi.ac.cy; b Department of Physics and NanoLund, Lund University 22100 Lund Sweden; c Chemical Engineering, Delft University 2629 HZ Delft The Netherlands; d Faculty of Civil Engineering and Geosciences, Delft University of Technology 2628 CN Delft The Netherlands

## Abstract

Nanoparticles (NPs) mixed at the atomic scale have been synthesized by atmospheric-pressure spark ablation using pairs of Pd and Hf electrodes. Gravimetric analysis of the electrodes showed that the fraction of each material in the resulting mixed NPs can be varied from *ca.* 15–85 at% to 85–15 at% by employing different combinations of electrode polarities and thicknesses. These results were also qualitatively corroborated by microscopy and elemental analysis of the produced NPs. When using pairs of electrodes having the same diameter, the material from the one at negative polarity was represented at a substantially higher fraction in the mixed NPs regardless of whether a pair of thin or thick electrodes were employed. This can be attributed to the higher ablation rate of the electrodes at the negative polarity, as already known from earlier experiments. When using electrodes of different diameters, the fraction of the element from the thinner electrode was always higher. This is because thinner electrodes are ablated more effectively due to, at least in part, the increased importance of the associated heat losses compared to its thicker counterpart. In those cases, the polarity of the electrodes had a significantly smaller effect. Overall, our results demonstrate, for the first time, that spark ablation can be used to control atomic scale mixing and thus produce alloyed NPs with compositions that can be tuned to a good extent by simply using different combinations of electrode diameters and polarities. This expands the capabilities of the technique for producing mixed nanoparticle building blocks of well-defined composition that are highly desired for a wide range of applications.

## Abstract 2

Mixed nanoparticles at atomic scale have been synthesized by atmospheric-pressure spark ablation using different combinations of electrode diameters–polarities.

## Graphical abstract

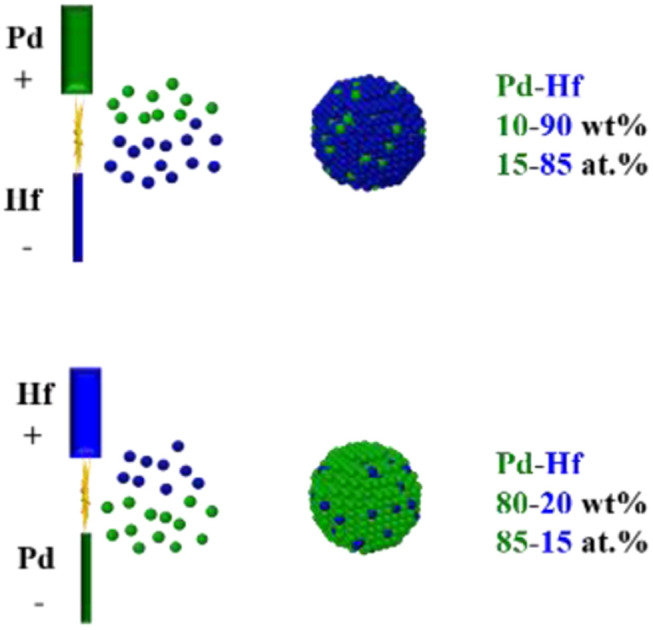

## 1. Introduction

Introduced in 1988 by Schwyn *et al.*,^1^ spark ablation is a gas-phase nanoparticle (NP) synthesis method that relies on the evaporation of bulk materials (in the form of electrodes) by inducing electric discharges between them.^2^ The material ablated by the electrodes forms a vapor cloud that is quenched and carried away by a gas flow, forming atomic clusters and NPs upon nucleation and growth. Spark ablation yields NPs of well-defined size and composition in a simple, inexpensive, and environmentally friendly manner as it does not require chemical precursors and avoids waste production.

A great advantage of the method is that it can allow mixing of different elements at the atomic and nanometer scales. Bimetallic NPs mixed at the atomic scale (*i.e.*, alloy NPs) can be produced using one spark discharge generator (SDG) and alloyed electrodes, or two electrodes of different elements.^3^ This has been shown even for combinations of materials that are immiscible in the bulk.^4^ Mixing at the nanometer scale (*e.g.*, yielding core–shell or decorated NPs) can be achieved using two SDGs (in series or in parallel), or, under specific conditions, one SDG and two electrodes of different elements that are immiscible,^5^ such as Cu and Ag. Combined with aerosol NP deposition systems including electrostatic precipitators, focusing impactors, and impactor-printing systems, the technique can produce a wide range of nanomaterials for catalysis, sensing or other applications.^6–8^

Mixed Pd-based NPs, which we use here to demonstrate the concept of tunable mixing by spark ablation, are of great interest because of their unique electronic, optical and catalytic properties, making them appropriate for a wide range of applications, including gas sensing and catalysis.^9–12^ In particular, Pd–M (where M can be Ir, Au, Ni, Zn, Ag, or Cu) alloy NPs have shown superior catalytic performance, in terms of activity and selectivity, compared to monometallic NPs for the reduction of NO by H_2_,^13–15^ for CO oxidation,^16^ and for hydrogenation of chloronitrobenzenes^17^ among others. Pd–Au NP-based films have also been shown to provide enhanced H_2_ optical sensing capabilities compared to their classical thin-film counterparts,^18^ whereas similarly-mixed NPs hold great promises as materials for environmental metal oxide semiconductor gas sensors.^19,20^

More specifically for gas sensing, Pd is considered one of the most effective elements for H_2_ sensors because it can easily form palladium hydride upon exposure to the gas, which exhibits altered electrical and optical properties.^21^ Considering that, Pd-based thin films have been extensively explored in optical H_2_ sensing,^22–25^ but their performance is hindered by hysteresis; a phenomenon whereby the optical behavior of the material with increasing H_2_ pressure differs from that when decreasing it, due to its transition from α-PdH_*x*_ to β-PdH_*x*_ phase at low and high H_2_ pressures, respectively. The hysteresis effect makes the Pd-based sensing materials problematic because their response does not only depend on the H_2_ pressure they are exposed to, but also on the history of their exposure to the target gas.^21,26^ This can be circumvented by alloying Pd with other metals, which has been shown to suppress the undesirable transition from α-PdH_*x*_ to β-PdH_*x*_, and consequently eliminate the unwanted hysteresis.^27–29^ For example, Pd–M (where M can be Ta or Au) alloy thin films were found to offer a hysteresis-free optical H_2_ sensing over an extremely wide range in hydrogen pressures.^30,31^ Apart from Pd-based materials, Hf thin films have also been shown to provide a family of promising optical H_2_-sensing materials,^32,33^ whereas Pd-capped Hf and Ta thin films show negligible hysteresis and high contrast.^33,34^

Considering the usefulness of mixed Pd–Hf materials, here we investigate the ability to produce their NP building blocks, in a tunable way, by spark ablation. Using different combinations of electrode polarities and thicknesses, we conducted systematic experiments to understand how their composition can vary and consequently be tuned/controlled. The rest of the paper is organized as follows. Section 2 provides details of our experimental methods, Section 3 presents and discusses our results, and section 4 highlights the most important conclusions.

## 2. Experimental

Mixed Pd–Hf NPs were synthesized by spark ablation using a commercial SDG (VSParticle, Model G1) with high-purity electrodes (99.95% for Pd and 97% for Hf) having diameters of 2 or 0.25 mm. The electrodes were placed coaxially next to each other within the SDG chamber, with a gap of 1 mm between them. A 2 L min^−1^ flow of pure (99.999% purity) N_2_ was used as a carrier gas, whereas the energy per spark and the sparking frequency in the SDG were set respectively to 22.5 mJ and 100 Hz.

**Fig. 1:**
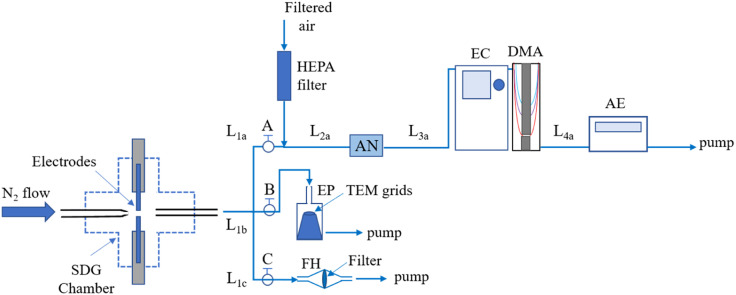
Schematic layout of the experimental set-up used in our study, including conceptual values A, B and C to describe the flow path during each experiment. For measurements of the size distributions of the NPs produced by the SDG, valve A was open and valve B and C were closed. To deposit the NPs on grids for the microscopy analysis, valve A and C were closed and valve B was open. To collect NPs on filters for XRD measurements, valve A and B were closed and valve C was open. The lengths of the tubes connecting all the experimental components were as follows: *L*_1a_ = *L*_1b_ = *L*_1c_ = 10 cm; *L*_2a_ = 15 cm; *L*_3a_ = 63 cm; and *L*_4a_ = 25 cm. We should note here that *L*_1b_ and *L*_1c_ correspond to the total tube length from the SDG to the EP or the FH, respectively, and not up to the conceptual valves A and B. Key: SDG: spark discharge generator; AN: aerosol neutralizer; EC: electrostatic classifier; DMA: differential mobility analyzer; AE: aerosol electrometer; EP: electrostatic precipitator; TEM grids: transmission electron microscope grids; FH: filter holder.

A Scanning Mobility Particle Sizer (SMPS) comprised of a Differential Mobility Analyzer (TSI, Model 3081) connected to an ^241^Am Aerosol Neutralizer (GRIMM, Model 5.522), an electrostatic classifier (TSI Model 3080), and an aerosol electrometer (TSI, Model 3068A) was used to measure the size distributions of the Pd–Hf NPs produced by the SDG (*cf.*Fig. 1). The aerosol and sheath flows in the DMA were set to 5 and 10 L min^−1^, respectively, whereas the voltage was scanned from 0.01 to 5.30 kV enabling the SMPS to record size distributions of the sampled NPs in the range of 7–207 nm. For all the measurements, the aerosol flow immediately downstream of the SDG was diluted by a factor of 2.5, which was taken into account when reporting the size distributions.

Gravimetric measurements of the electrodes were carried out in order to estimate the ablated electrode mass using a high accuracy balance (KERN Model ABT 100-5MA). The mass of each electrode was measured before and after using the SDG for 2 h to produce mixed particles, and the measurements were used to determine the fraction of ablated mass from each electrode. The mean masses ablated from each electrode, and the associated standard deviations, were calculated from 12 different experiments carried out under the same conditions.

A custom-made electrostatic precipitator (Fig. 1) was used to deposit the NPs on Transmission Electron Microscope grids (Ted Pella, Prod. No. 01890), whereas a custom-made filter holder was used to collect NPs, in the form of powder samples, for X-ray diffraction (XRD) measurements. The distance from the SDG to the electrostatic precipitator or the filter holder was 10 cm (Fig. 1), and the deposition of the NPs for microscopy analysis was set to 10 min in all cases. We should note here that due to the high concentration of particles downstream of the SDG, they grew in size by coagulation, leading to agglomerated structures as shown in the microscopy images provided in the next session.

XRD analysis of powder samples compromised of Pd or Pd–Hf NPs were recorded using a MiniFlex 600 benchtop diffractometer (Rigaku; CuKα radiation, *λ* = 1.5418 Å). For these measurements the samples were put under an Ar flow (1 L min^−1^) at ambient temperature, whereas the diffraction angle 2*θ* ranged from 10 to 90° with a scaning speed of 10° min^−1^.

The nanoparticles were further characterized using a Transmission Electron Microscope (TEM; Jeol JEM-3000F) with scanning/transmission operating modes (TEM/STEM). High-resolution (HR) TEM was used to study the agglomeration and crystallinity of the particles. An Energy-Dispersive X-ray Spectrometer (EDS; Oxford Instruments, X-Max 80 mm^2^ Silicon Drift Detector, SDD) coupled to the TEM was employed to study the elemental composition of the particles. HR-TEM-EDS mode was used to obtain the chemical composition of agglomerates, *i.e.*, ensembles of primary particles. In addition to that, Scanning TEM (STEM)-EDS mode was used to determine the elemental composition of individual primary particles. For each sample, *i.e.*, electrode configuration, a total of at least 20 measurements were carried out in each TEM mode. The HR-TEM micrographs were analyzed with ImageJ,^35^ whereas the EDS data was evaluated with AZtec and INCA software.

## 3. Results and discussion

**Fig. 2:**
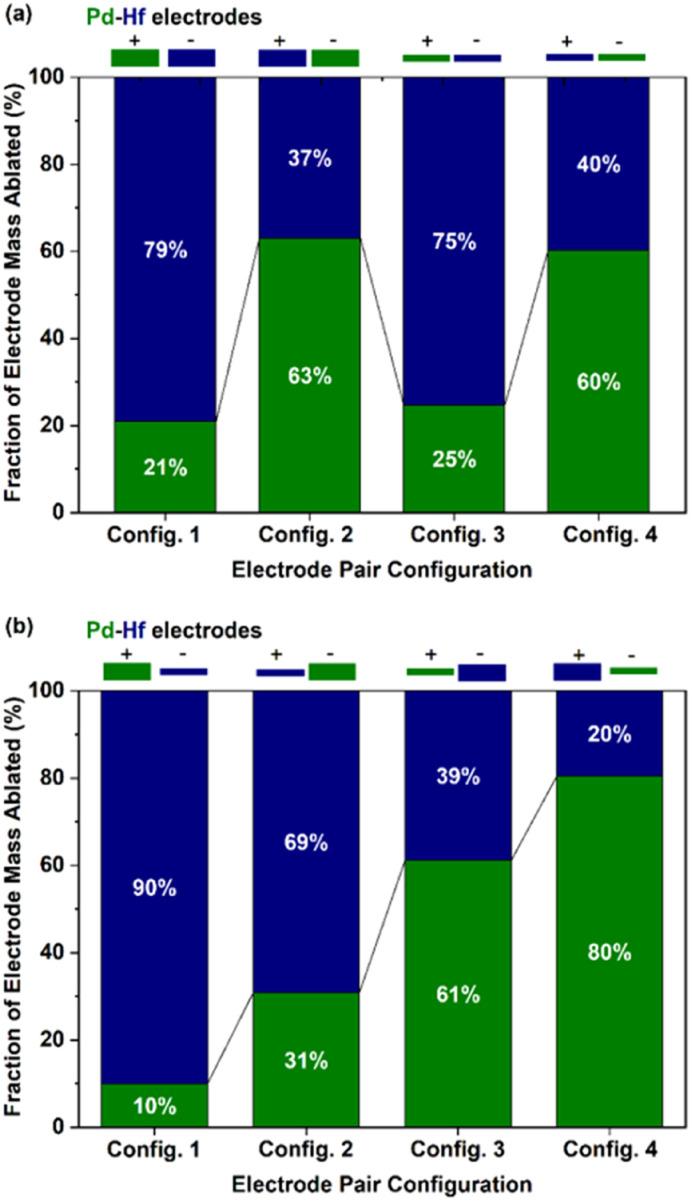
Fraction of masses (wt%) ablated by the Pd (green part of the bars) and Hf (blue part of the bars) electrode configurations of (a) different polarity but of the same thickness, and (b) different polarity and thickness. In all cases, the thick and thin electrodes had diameters of 2 and 0.25 mm, respectively.

Fig. 2 shows the results from the gravimetric measurements described above. Fig. 2a shows the fraction of material ablated (wt%) from each electrode (Pd and Hf) when their thickness was the same, but their polarity was switched. In all cases, the negative electrode produced more material than the positive, independently from their thickness. According to Tabrizi *et al.*,^3^ this is expected because the positive ions hitting the negative electrode are heavier than their negative counterparts, which are produced by impurities in the carrier gas and electrons. As a result, the energy transferred to the negative electrode is higher compared to that transferred to the positive, resulting in more intensive ablation of the former. Evidently, the thickness of the electrodes employed did not significantly affect the fraction of masses ablated from each of them, as shown by comparing configs. 1 and 3 or configs. 2 and 4 in Fig. 2a.

Fig. 2b shows the fraction of the ablated mass (wt%) from each electrode when configurations of different diameters and polarity were used. Evidently, the fraction of ablated mass increases with the following order of electrode thickness–polarity combinations: thick-positive, thick-negative, thin-positive, and thin-negative electrodes. The higher ablation from thin electrodes can be attributed in part to the lower heat losses of the thinner electrodes,^36^ or to the field focusing effect.^2^ Overall, the fraction of ablated electrode mass for the Pd–Hf system was varied from *ca.* 10–90 to 80–20 wt% for the eight electrode configurations we used (*cf.* results in Fig. 2a and b). Expressed as at%, the results from gravimetric measurements shown in Fig. 2a are provided in Fig. S1 in the ESI,† whereas the results shown in Fig. 2b are discussed further below (*cf.*Fig. 6 and associated discussion).

**Fig. 3:**
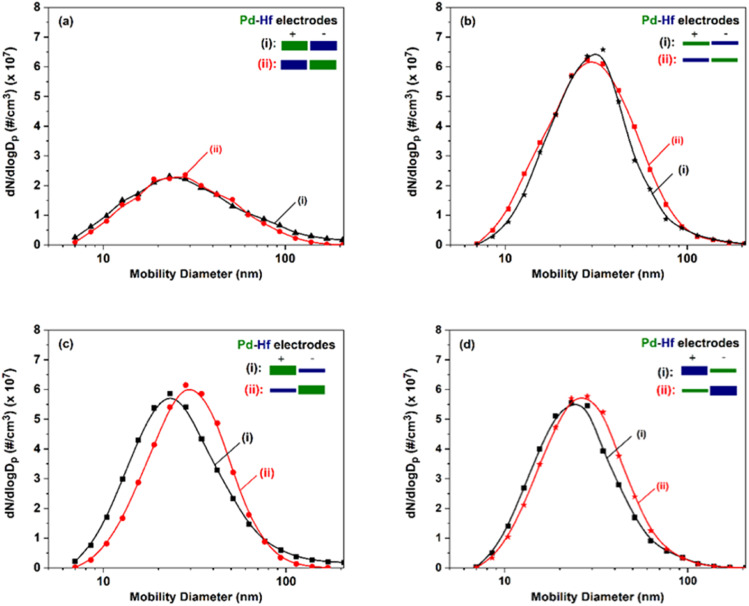
Particle size distributions, measured by the SMPS, of the Pd–Hf NPs produced by the SDG when using pairs of (a) thick electrodes, (b) thin electrodes, (c) a thin Hf and a thick Pd electrode, and (d) a thin Pd and a thick Hf electrode. For each case, comparison of the size distributions is made when switching the polarity of the electrode pairs. The thick and thin electrodes used in our measurements had diameters of 2 and 0.25 mm, respectively.

Fig. 3 shows particle size distributions of the agglomerated Pd–Hf NPs produced by the SDG as recorded by the SMPS. The total number concentration increased by a factor of more than 2, *i.e.*, from 1.7 × 10^7^ to 3.7 × 10^7^ #/cc, whereas the mean particle size increased marginally, from *ca.* 24 to 30 nm, when switching from thick to thin electrode pairs (*cf.*Fig. 3a and b). When using electrode pairs of different thicknesses and polarities (*cf.*Fig. 3c and d), the concentration of the particles remained in the same range (3.1–3.5 × 10^7^ #/cc) while the size of agglomerated NPs varied from 25 to 30 nm. These results corroborate previous reports showing that thinner electrodes increase NP throughput in SDGs,^36^ and indicate that the resulting concentrations remain in the same range as long as one of the electrodes is thin regardless of its polarity.

**Fig. 4:**
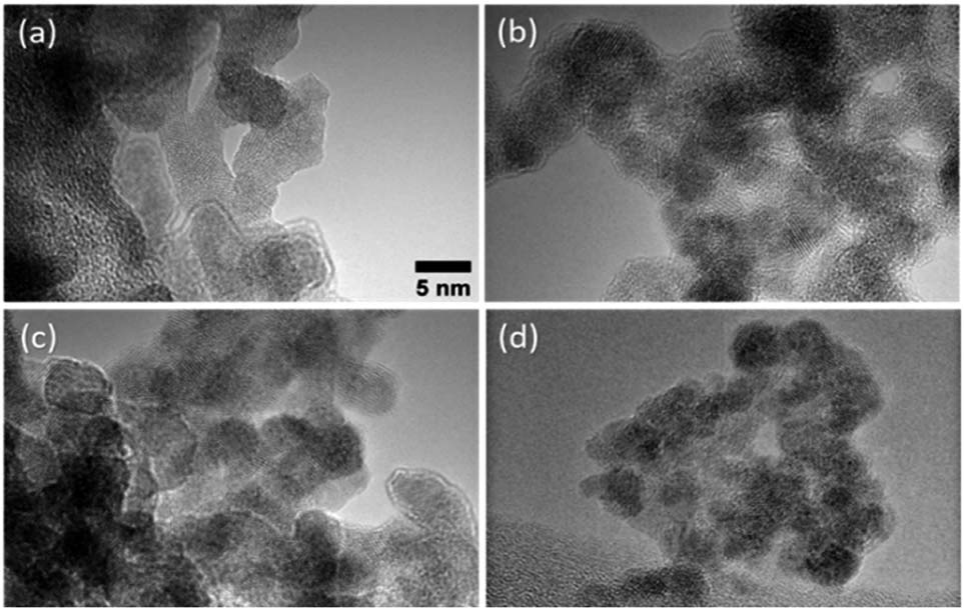
TEM images of Pd–Hf agglomerates produced by the SDG when using the following electrode configurations: (a) a thick-positive Pd and a thin-negative Hf electrode, (b) a thick-negative Pd and a thin-positive Hf electrode, (c) a thin-positive Pd and a thick-negative Hf electrode, and (d) a thin-negative Pd and a thick-positive Hf electrode. In all cases, the thick and thin electrodes had diameters of 2 and 0.25 mm, respectively.

TEM images of the agglomerated Pd–Hf particles produced by the SDG are provided in Fig. 4, showing that they consist of primary particles having sizes of *ca.* 5 nm. This is in line with results reported by Feng *et al.*^37^ indicating that the size of primary particles increases to a critical size, above which coalescence only partly occurs or ceases, thus leading to the formation of non-spherical fractal-like agglomerates.

**Fig. 5:**
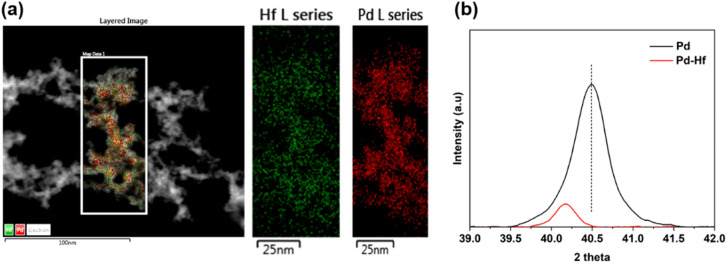
(a) STEM-EDS mapping of Pd–Hf NP samples synthesized using thin-negative Pd and thick-positive Hf electrodes. (b) XRD of pure Pd and Pd–Hf NP-containing samples (in the form of powders) and recorded under Ar flow (1 L min^−1^) at 35 °C. The Pd NP-containing sample was synthesized using two thick (2 mm in diameter) Pd electrodes, whereas the Pd–Hf sample using two thick electrodes (2 mm in diameter): *i.e.*, a negative Pd and a positive Hf electrode.

The TEM analysis also indicates that the Pd–Hf NPs formed are alloyed as atoms of Pd and Hf are uniformly distributed in the samples (*cf.*Fig. 5a). We should note here that Pd and Hf have similar atomic radii (*i.e.*, 0.78 Å for Pd and 0.85 Å for Hf) and surface energy (*i.e.*, 1.9 J m^−2^ for Pd and 2.4 J m^−2^ for Hf),^38^ and can therefore easily form a homogeneous solid solution with a negative formation enthalpy (exothermic alloying process),^39^ supporting the microscopy observations reported here.

The XRD measurements of the Pd and Pd–Hf NP samples shown in Fig. 5b also support that the latter are alloyed. This is because the peak in the Pd–Hf XRD spectrum shifts to a lower 2*θ* value compared to that of pure Pd, suggesting expansion of Pd lattice due to the introduction of Hf, which has a slightly larger atomic radius compared to Pd (*cf*. values provided above). In addition, Selected Area electron diffraction (SAED) results show crystalline regions embedded in amorphous phases (*cf*. Fig. S2 in ESI†).

**Fig. 6:**
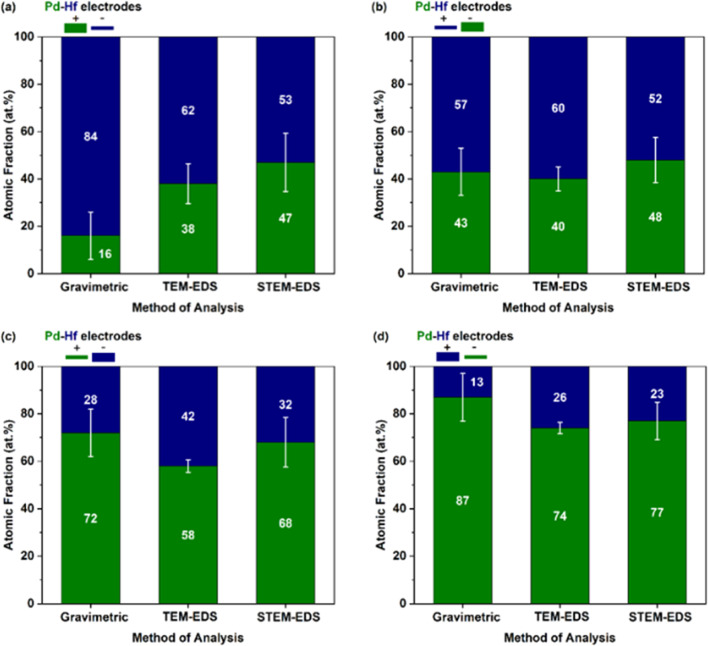
Fraction of Pd (green parts of the bars) and Hf (blue parts of the bars), expressed as at%, determined by gravimetric, TEM-EDS, and STEM-EDS analysis for different electrode configurations: (a) a thick-positive Pd and a thin-negative Hf electrode, (b) a thick-negative Pd and a thin-positive Hf electrode, (c) a thin-positive Pd and a thick-negative Hf electrode, and (d) a thin-negative Pd and a thick-positive Hf electrode.

The composition of the agglomerated and primary Pd–Hf mixed NPs was also determined by TEM-EDS and STEM-EDS analysis, respectively. The at% fraction of each element in the agglomerated and primary mixed NPs is provided in Fig. 6, together with the gravimetric results described above but now converted to at% to serve the comparison. We should note here that the composition results from the TEM/STEM-EDS measurements reported here correspond to both the crystalline and amorphous regions of the sampled NPs, and are averages from at least 20 measurements with the error bars indicating one standard deviation.

Evidently, the results from the TEM-EDS, STEM-EDS and gravimetric measurements compared in Fig. 6 show the same overall trend on how the composition of the NPs changes with the different configurations investigated. For some configurations (*e.g.*, for the positive-thin Hf and negative-thick Pd electrode; *cf.*Fig. 6b), the agreement of the three measurement approaches is very good. We should highlight here that the gravimetric measurements can be biased by the production of splashing particles, (*i.e.*, particles larger than ∼100 nm that form by the ejection of droplets from molten pools formed locally by individual sparks).^2^ Production of splashing particles depends on a number of factors, including the properties of the electrode material and its thickness, and thus agreement with the composition measurements determined by individual particles with the TEM-EDS or the STEM-EDS can exhibit deviations. The good overall agreement of the gravimetric with the TEM/STEM-EDS results observed here indicates that the amounts of splashing particles in our measurements ranged from small to negligible. This was corroborated by the microscopy analysis that showed for some samples only a small number of splashing particles consisting of one or the other metal (*i.e.*, Pd or Hf; data now shown).

**Fig. 7:**
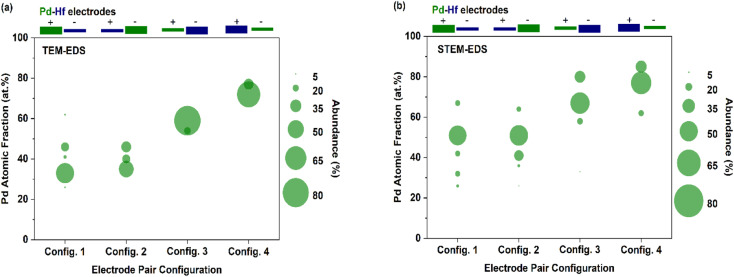
Fraction of Pd (at%) in the mixed, (a) agglomerated (determined by TEM-EDS), and (b) primary (determined by STEM-EDS) particles produced by the SDG when using different electrode pair configurations. In all cases, the thick and thin electrodes had diameters of 2 and 0.25 mm, respectively.

Interestingly, the composition of Pd–Hf alloy NPs was not uniform but varied from case to case in all the samples. Fig. 7 shows the fraction Pd (expressed as at%) in the agglomerated (Fig. 7a, TEM-EDS) and primary (Fig. 7b, STEM-EDS) particles, whereas the raw data of these measurements are provided in Tables S1–S5.† For configurations 1 and 2 (Fig. 7a) where the Pd electrode was thick, the fraction of the material varied from 30 to 60 at%, with the most abundant composition being between 30 and 35 at%. For configurations 3 and 4 (Fig. 7a) where the Pd electrodes were thin, the composition of agglomerated particles was 60 and 75 at%, with the latter being higher because the polarity of the electrode was negative.

Fig. 7b shows the fraction of Pd (at%) in the primary particles as determined by STEM-EDS analysis. Interestingly, the variability of the composition of the primary particles was higher than that of the agglomerates (*i.e.*, as shown in Fig. 7a), which is expected because of the high resolution of the STEM-EDS measurements. More specifically, three to five distinct compositions of individual primary particles were observed for each electrode configuration. In configurations 1 and 2, the Pd atomic fraction in the resulting primary NPs varied from 25 to 70 at%, with the most abundant species containing 50 at% of the element. Configurations 3 and 4 had higher overall Pd atomic fractions with the most abundant species having values of *ca.* 70 and 80 at%, respectively. Despite the high variability, these measurements show that the overall composition of the primary particles can be tuned to a good extent by using different electrode diameter-polarity configurations, demonstrating that spark ablation can be used to control nanoparticle mixing at the atomic scale.

## 4. Conclusions

Pd–Hf NPs mixed at the atomic scale were synthesized by atmospheric-pressure spark ablation using pairs of electrodes of the two elements, *i.e.*, Pd and Hf. Overall, the composition of the resulting mixed Pd–Hf NPs varied from 15–85 to 85–15 at%, depending on the combination of the diameters and polarity of each electrode. The electrodes set on negative polarity produced higher fractions of material in the resulting NPs when the two electrodes in each pair had the same thickness. When the thickness of the electrodes was also varied, the thinner ones contributed more to the resulting mixed NPs, independently of their polarity. Taken together, the fraction each element in the resulting mixed NPs increased with the following order of electrode thickness–polarity combinations: thick-positive, thick-negative, thin-positive, and thin-negative electrodes. The findings of this study expand our knowledge on mixed NP production by spark ablation, demonstrating for the first time the ability of the technique to control mixing at the atomic scale and consequently to tune the composition of the resulting particles over a wide range, extending considerably its potentials.

## Author contributions

Klito C. Petallidou: validation, investigation, visualization, writing – original draft. Pau Ternero: validation, investigation, writing – review & editing. Maria E. Messing: writing – review & editing. Andreas Schmidt-Ott: supervision, writing – review & editing. George Biskos: conceptualization, methodology, supervision, project administration, writing – review & editing.

## Conflicts of interest

There are no conflicts to declare.

## Supplementary Material

NA-005-D3NA00152K-s001
