# Supplementary material for: Tuning atomic-scale mixing of nanoparticles produced by atmospheric-pressure spark ablation
Source: Nanoscale Adv. 2023 Aug 23;5(24):6880–6. doi: 10.1039/d3na00152k (PMC10697000; doi:10.1039/d3na00152k)
Supplement: NA-005-D3NA00152K-s001 [file NA-005-D3NA00152K-s001.pdf]

Electronic Supplementary Material (ESI) for Nanoscale Advances.
This journal is © The Royal Society of Chemistry 2023

Electronic Supplementary Information

Tuning atomic-scale mixing of Pd-Hf nanoparticles by atmospheric-pressure spark ablation

Klito C. Petallidou[a], Pau Ternero[b], Maria E. Messing[b],

Andreas Schmidt-Ott[a,c] and George Biskos*[a,d]

[a]Climate and Atmosphere Research Centre, The Cyprus Institute, 2121 Nicosia, Cyprus
[b]Department of Physics and NanoLund, Lund University, 22100 Lund, Sweden
[c]Chemical Engineering, Delft University, 2629 HZ Delft, The Netherlands
[d]Faculty of Civil Engineering and Geosciences, Delft University of Technology, 2628 CN Delft, The Netherlands

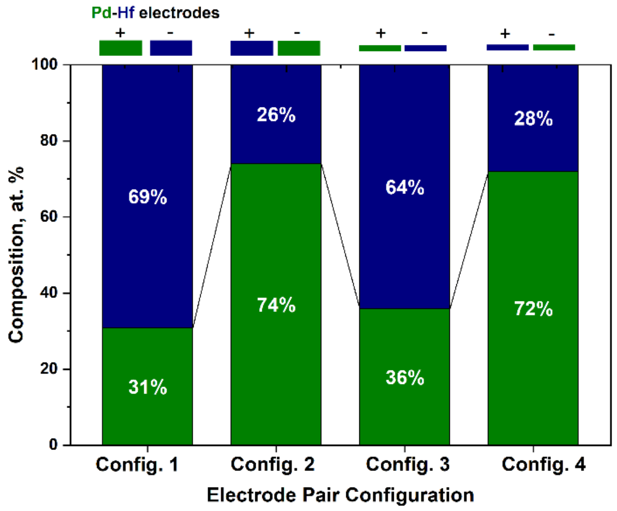

Figure S1. Fraction of Pd (green) and Hf (blue), estimated by the corresponding at.%, determined by gravimetric analysis for different electrode configurations, different polarity but of the same thickness In all cases, the thick and thin electrodes had diameters of 2 and 0.25 mm, respectively.

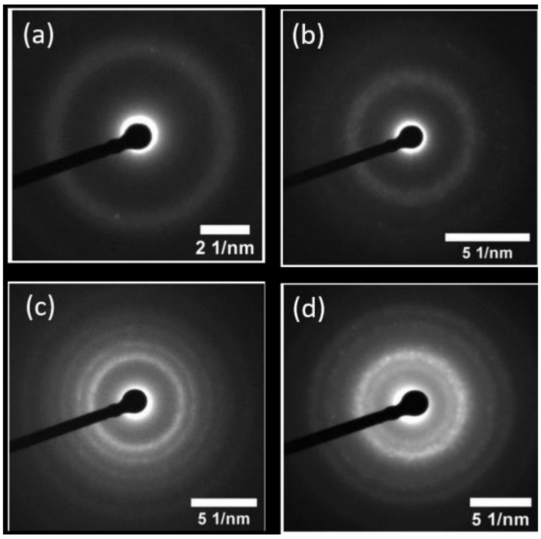

Figure S2. SAED patterns for different electrode configurations (a) Configuration 1: Pd (2 mm, positive) and Hf (0.25 mm, negative); (b) Configuration 2: Pd (2 mm, negative) and Hf (0.25 mm, positive); (c) Configuration 3: Pd (0.25 mm, positive) and Hf (2 mm, negative); (d) Configuration 4: Pd (0.25 mm, positive) and Hf (2mm, negative).

Table S1. Pd:Hf composition (at. %) estimated from TEM-EDS for different electrode configurations, different polarity and thickness. **Configuration 1**: Pd (2 mm, positive) and Hf (0.25 mm, negative); **Configuration 2**: Pd (2 mm, negative) and Hf (0.25 mm, positive); **Configuration 3**: Pd (0.25 mm, positive) and Hf (2 mm, negative); **Configuration 4**: Pd (0.25 mm, positive) and Hf (2mm, negative).

**Spectrum**	**Configuration 1**		**Configuration 2**		**Configuration 3**		**Configuration 4**	
	**Pd (at.%)**	**Hf (at.%)**	**Pd (at.%)**	**Hf (at.%)**	**Pd (at.%)**	**Hf (at.%)**	**Pd (at.%)**	**Hf (at.%)**
**1**	46.32	53.68	44.72	55.28	57.92	42.08	75.77	24.23
**2**	44.94	55.06	37.53	62.47	58.94	41.06	78.46	21.54
**3**	46.08	53.92	48.61	51.39	57.61	42.39	73.35	26.65
**4**	47.38	52.62	48.40	51.60	57.00	43.00	73.67	26.33
**5**	61.57	38.43	35.73	64.27	61.00	39.00	72.67	27.33
**6**	34.00	66.00	33.03	66.97	57.20	42.80	76.92	23.08
**7**	41.42	58.58	36.15	63.85	55.08	44.92	73.31	26.69
**8**	45.71	54.29	44.96	55.04	58.25	41.75	71.40	28.60
**9**	41.50	58.50	40.17	59.83	63.34	36.66	74.38	25.62
**10**	33.18	66.82	36.36	63.64	62.28	37.72	71.91	28.09
**11**	34.01	65.99	41.22	58.78	57.87	42.13	77.94	22.06
**12**	29.61	70.39	44.67	55.33	54.01	45.99	76.43	23.57
**13**	33.55	66.45	38.73	61.27	59.11	40.89	73.37	26.63
**14**	38.47	61.53	46.47	53.53	53.65	46.35	68.97	31.03
**15**	29.73	70.27	42.91	57.09	60.14	39.86	72.12	27.88
**16**	31.15	68.85	36.14	63.86	60.86	39.14	76.50	23.50
**17**	26.40	73.60	33.81	66.19	59.22	40.78	71.09	28.91
**18**	31.86	68.14	33.78	66.22	54.82	45.18	74.08	25.92
**19**	35.03	64.97	34.07	65.93	61.86	38.14	72.12	27.88
**20**	37.80	62.20	40.87	59.13	56.12	43.88	73.16	26.84

Table S2. Pd:Hf composition (at. %) estimated from STEM-EDS for different electrode configurations, different polarity and thickness. **Configuration 1**: Pd (2 mm, positive) and Hf (0.25 mm, negative);

**Spectrum**	**Pd (at.%)**	**Hf (at.%)**

**1**	40.03	59.97
**2**	41.56	58.44
**3**	55.74	44.26
**4**	57.10	42.90
**5**	71.62	28.38
**6**	48.09	51.91
**7**	55.88	44.12
**8**	49.20	50.80
**9**	33.49	66.51
**10**	26.12	73.88
**11**	45.41	54.59
**12**	56.29	43.71
**13**	36.43	63.57
**14**	50.22	49.78
**15**	55.18	44.82
**16**	49.58	50.42
**17**	50.45	49.55
**18**	47.00	53.00
**19**	41.88	58.12
**20**	51.87	48.13
**21**	52.55	47.45
**22**	50.11	49.89
**23**	67.61	32.39
**24**	67.00	33.00
**25**	44.34	55.66
**26**	29.06	70.94
**27**	59.36	40.64

**28**	27.69	72.31
**29**	28.38	71.62
**30**	41.53	58.47
**31**	67.55	32.45
**32**	47.60	52.40
**33**	35.24	64.76
**34**	23.59	76.41
**35**	43.72	56.28

Table S3. Pd:Hf composition (at. %) estimated from STEM-EDS for different electrode configurations, different polarity and thickness. **Configuration 2**: Pd (2 mm, negative) and Hf (0.25 mm, positive)

**Spectrum**	**Pd (at.%)**	**Hf (at.%)**
**1**	40.43	59.57
**2**	41.17	58.83
**3**	46.56	53.44
**4**	53.03	46.97
**5**	66.79	33.21
**6**	25.86	74.14
**7**	43.23	56.77
**8**	37.45	62.55
**9**	35.51	64.49
**10**	48.48	51.52
**11**	54.65	45.35
**12**	40.93	59.07
**13**	52.49	47.51
**14**	51.87	48.13
**15**	41.50	58.50
**16**	58.50	41.50

**17**	52.01	47.99
**18**	48.85	51.15
**19**	34.35	65.65
**20**	56.31	43.69
**21**	61.12	38.88
**22**	39.91	60.09
**23**	54.78	45.22
**24**	49.26	50.74
**25**	45.43	54.57
**26**	41.32	58.68
**27**	38.63	61.37
**28**	64.23	35.77
**29**	48.32	51.68
**30**	39.10	60.90
**31**	41.23	58.77
**32**	49.88	50.12
**33**	73.48	26.52
**34**	53.58	46.42
**35**	50.39	49.61
**36**	48.40	51.60
**37**	50.59	49.41
**38**	55.36	44.64
**39**	43.58	56.42
**40**	51.87	48.13
**41**	61.58	38.42
**42**	52.74	47.26
**43**	42.71	57.29

**44**	52.14	47.86

Table S4. Pd:Hf composition (at. %) estimated from STEM-EDS for different electrode configurations, different polarity and thickness. **Configuration 3**: Pd (0.25 mm, positive) and Hf (2 mm, negative)

Spectrum	Pd (at.%)	Hf (at.%)
**1**	64.35	35.65
**2**	71.14	28.86
**3**	68.77	31.23
**4**	58.22	41.78
**5**	78.22	21.78
**6**	57.00	43.00
**7**	79.63	20.37
**8**	58.68	41.32
**9**	64.87	35.13
**10**	66.42	33.58
**11**	81.68	18.32
**12**	76.16	23.84
**13**	81.34	18.66
**14**	59.37	40.63
**15**	70.57	29.43
**16**	65.34	34.66
**17**	68.57	31.43
**18**	65.51	34.49
**19**	82.51	17.49
**20**	64.63	35.37
**21**	67.03	32.97
**22**	62.67	37.33
**23**	68.36	31.64

**24**	75.33	24.67
**25**	62.62	37.38
**26**	79.29	20.71
**27**	80.02	19.98
**28**	56.07	43.93
**29**	32.67	67.33
**30**	81.74	18.26
**31**	64.55	35.45

Table S5. Pd:Hf composition (at. %) estimated from STEM-EDS for different electrode configurations, different polarity and thickness. **Configuration 4**: Pd (0.25 mm, positive) and Hf (2mm, negative).

Spectrum	Pd (at.%)	Hf (at.%)
**1**	84.51	15.49
**2**	74.14	25.86
**3**	75.29	24.71
**4**	61.47	38.53
**5**	79.47	20.53
**6**	85.24	14.76
**7**	79.11	20.89
**8**	84.27	15.73
**9**	87.52	12.48
**10**	75.14	24.86
**11**	76.20	23.80
**12**	60.84	39.16
**13**	78.81	21.19
**14**	77.76	22.24
**15**	73.03	26.97
**16**	83.12	16.88

**17**	63.24	36.76
**18**	78.26	21.74
**19**	80.79	19.21
**20**	87.33	12.67
